# Detecting Anomaly Event in Video Based on Generative Adversarial Network

**DOI:** 10.1155/2022/8633955

**Published:** 2022-10-05

**Authors:** Zhaoxian Zhang

**Affiliations:** Guilin University of Electronic Technology School of Information and Communication, Guangxi, Guilin 541000, China

## Abstract

Anomaly detection in videos is a challenging computer vision problem. Existing state-of-the-art video anomaly detection methods mainly focus on the structural design of deep neural networks to obtain performance improvements. Different from the main research trend, this paper focuses on combining ensemble learning and deep neural networks and proposes an approach based on ensemble generative adversarial network (GAN). In the proposed method, a set of generators and a set of discriminators are trained together, so each generator gets feedback from multiple discriminators and vice versa. Compared with a single GAN, the proposed ensemble GAN can better model the distribution of normal data to better detect anomalies. In the experiments, the performance of the proposed method is tested on two public datasets. The results show that ensemble learning significantly improves the performance of a single detection model, which outperforms some existing state-of-the-art methods.

## 1. Introduction

Anomaly detection in surveillance video is a fundamental computer vision task that plays a crucial role in video analysis. It can be well used in potential applications such as accident prediction, urban traffic analysis, and evidence investigation. Although the problem has attracted intense attention in recent years, video anomaly detection is still a very challenging work due to the severe imbalance between normal and anomalous samples, the lack of detailed anomaly labeled data, and the inconsistent definitions of anomalous behaviors.

To address this problem, researchers have proposed a number of methods. According to the literature review [1], existing anomaly detection methods can be divided into ones based on density estimation and probabilistic models, ones based on single-class classification, and ones based on reconstruction. The methods based on density estimation and probability model [2, 3] mainly calculate the probability density function of the samples at first and then make the judgement by obtaining the distance between the sample and the center of the density function. While the classical nonparametric density estimators perform reasonably well when dealing with low-dimensional problems, the sample size they require to achieve a fixed level of accuracy grows exponentially in the dimension of the feature space. One-class classification-based methods [4, 5] try to avoid full estimation of density as an intermediate step in anomaly detection, and these methods aim to directly learn the decision boundary corresponding to the positive samples, by testing whether the samples under test are within the boundary. Reconstruction-based methods [6, 7] learn a model that is optimized to reconstruct normal data instances well, thereby detecting anomalies by failing to reconstruct them accurately under the learned model.

In recent years, the deep learning models learn efficient representations from the multiple sources of data by training flexible multi-layer deep neural networks, which has achieved breakthroughs in many applications involving complex data types, such as computer vision [8, 9], speech recognition [10, 11], or natural language processing [12, 13]. Methods based on deep neural networks are able to exploit the often inherent hierarchical or latent structure of data through their multi-layer distributed feature representations. Furthermore, advances in parallel computing, stochastic gradient descent optimization, and automatic differentiation have made it possible to apply deep learning at scale on large datasets. For anomaly detection problems, deep learning methods can optimize the entire anomaly detection model end-to-end and can also learn representations specifically for the anomaly detection problem. In addition, the ability of deep learning methods for large datasets helps to greatly improve the utilization of labeled normal data or some labeled anomalous data.

Under the framework of deep learning, this paper proposes an anomaly detection method based on single-class classification. This approach is an improved form of generative adversarial network (GAN) called GAN ensembles. GAN exploits the competition between the generator and the discriminator, where the generator learns the distribution of samples and the discriminator learns how to detect anomalies. An ensemble GAN consists of multiple encoder-decoders and discriminators that are randomly paired and trained via adversarial training. In this process, the encoder-decoder gets feedback from multiple discriminators, and the discriminator gets “training samples” from multiple generators. Compared with a single GAN, the proposed ensemble GAN can better model the distribution of normal data so it can be better employed to detect anomalies. Finally, the total anomaly score is obtained by taking the average of the calculated anomaly scores from all encoder-decoder discriminator pairs for discrimination. Experimental results on two public benchmark datasets show that the proposed method significantly outperforms some existing methods on a range of anomaly detection tasks.

## 2. Basic Principle

### 2.1. Description of Problem

**Figure 1:**
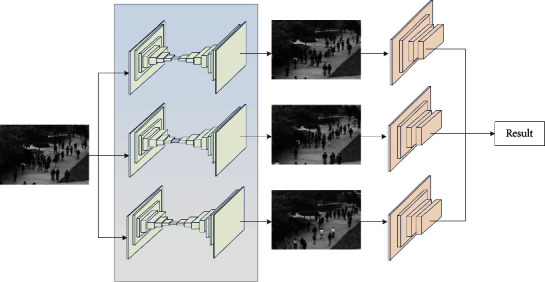
The methodology of abnormal event detection based on GAN ensembles.

Assuming that the normal sample training set is **X**={**x**_*i*_ ∈ *ℝ*^*d*^ : *i*=1,…, *N*}, which contains *N* samples from an unknown distribution *𝒟*, the sample **x**′ ∈ *ℝ*^*d*^ to be tested may not belong to the unknown distribution *𝒟*. Then, the problem of anomaly detection is to train the model from **X** such that the model can classify **x**′ as a normal sample if **x**′ belongs to the unknown distribution *𝒟*. Conversely, it is anomalous if **x**′ comes from a different distribution. Typically, the model computes the anomaly score *y*′ ∈ *ℝ*^*d*^ of **x**′ and determines the label of **x**′ by thresholding *y*′.  Figure 1 shows the general framework of the proposed method.

### 2.2. GAN

A typical GAN consists of two neural networks, i.e., a generator and a discriminator. Among them, the generator contains an encoder *G*_*e*_(·; *ϕ*) and a decoder *G*_*d*_(·; *ψ*). The encoder encodes the sample *x* into a vector *z*, and the decoder reconstructs it into vector $\tilde{x}$. The basic process is as follows:

(1) $$\begin{array}{c} z=G_{e}\left(x;\phi \right)\text{,}\\ \tilde{x}=G_{d}\left(z;\psi \right)\text{.} \end{array}$$

The discriminator *D*(·; *γ*) judges the probability that the test sample comes from the dataset **X** rather than the generator generated samples. Then, the discriminator should provide higher reconstruction error values for normal samples. Since the model consists of an encoder-decoder and a discriminator, the training process usually takes into account loss functions inherited from both models. The adversarial loss coming from GAN training is defined as follows:

(2) $$\begin{array}{c} L_{a-g}\left(x\right)=\log D\left(x\right)+\log\left(1-D\left(G_{d}\left(G_{e}\left(x\right)\right)\right)\right)\text{.} \end{array}$$

Another one is the reconstruction loss, which is used to train the encoder and decoder. In fact, the difference between the original sample and the reconstruction result is often calculated by the *l*-norm as follows:

(3) $$\begin{array}{c} L_{r}\left(x\right)={\left\|x-G_{d}\left(G_{e}\left(x\right)\right)\right\|}_{l}^{l}\text{.} \end{array}$$

Previous studies have shown that the hidden vector **h** of a sample in the last hidden layer of the discriminator *D*(·; *γ*) is useful for distinguishing normal samples from abnormal samples. Define *h*=*D*(**x**; *γ*) as the hidden vector in *D*(·; *γ*); then, the discriminant loss based on *h* can be calculated as follows:

(4) $$\begin{array}{c} L_{d}\left(x\right)={\left\|f_{D}\left(x\right)-f_{D}\left(G_{d}\left(G_{e}\left(x\right)\right)\right)\right\|}_{l}^{l}\text{.} \end{array}$$

Furthermore, GAN also considers the difference between the encoded vector of a normal sample **x** and its reconstruction $\tilde{x}$. In particular, it encodes the reconstructed $\tilde{x}$ using a separate encoder $G_{e}\left(\cdot ;\tilde{\phi }\right)$. Then, the encoding loss is as follows:

(5) $$\begin{array}{c} L_{e}\left(x\right)={\left\|G_{e}\left(x;\phi \right)-G_{e}\left(G_{d}\left(G_{e}\left(x;\phi \right);\tilde{\phi }\right)\right)\right\|}_{l}^{l}\text{.} \end{array}$$

In (5), the encoder parameters *ϕ* and $\tilde{\phi }$ are distinctly different. To train the discriminator, the GAN model needs to maximize the adversarial loss, which is defined as follows:

(6) $$\begin{array}{c} \max_{\gamma }\overset{N}{\underset{i=1}{\sum }}L_{a}\left(x_{i};\phi ,\psi ,\gamma \right)\text{.} \end{array}$$

After the GAN parameters are trained, the anomaly score *A*(**x**′) needs to be calculated for the test sample **x**′. Then, the anomaly score is obtained by calculating the weighted sum of the reconstruction loss and the discriminant loss as follows:

(7) $$\begin{array}{c} A\left(x^{\prime }\right)=L_{r}\left(x^{\prime }\right)+\beta L_{d}\left(x^{\prime }\right)\text{.} \end{array}$$

In (7), the weight *β* is obtained through empirical selection. A higher anomaly score indicates a high anomaly probability.

### 2.3. Anomaly Detection Based on GAN

This paper proposes an anomaly detection method based on ensemble GANs. The model contains multiple generators and discriminators, with different parameterizations. Assuming that *I* generators {*G*_*e*_(·; *ϕ*_*i*_), *G*_*d*_(·; *ψ*_*i*_) : *i*=1, ⋯, *I*} and *J* discriminators {*D*_*e*_(·; *γ*_*i*_), :*j*=1, ⋯, *J*} are defined, a single generator or discriminator is the same as the base model. During the adversarial training, each generator is matched with each discriminator, which is then evaluated by each discriminator. Also, the discriminator receives synthetic samples from each generator.

For multiple pairs of generators and discriminators, both adversarial and discriminative losses are computed from all generator-discriminator pairs. The loss between each generator-discriminator pair is calculated as follows:

(8) $$\begin{array}{c} L_{a}^{ij}=L_{a}\left(x;\phi_{i},\psi_{i},\gamma_{j}\right)\text{,}\\ L_{d}^{ij}=L_{d}\left(x;\phi_{i},\psi_{i},\gamma_{j}\right)\text{.} \end{array}$$

Similarly, the reconstruction loss and encoding loss for a single generator are calculated as follows:

(9) $$\begin{array}{c} L_{r}^{i}=L_{r}\left(x;\phi_{i},\psi_{i}\right)\text{,}\\ L_{e}^{i}=L_{r}\left(x;\phi_{i},\psi_{i}\right)\text{.} \end{array}$$

The discriminator is then trained by maximizing the sum of adversarial losses, while the generator is trained by minimizing the sum of all losses. The objective function is as follows:

(10) $$\begin{array}{c} \max_{{\left(\gamma_{j}\right)}_{j=1}^{J}}\overset{I}{\underset{i=1}{\sum }}\overset{J}{\underset{j=1}{\sum }}L_{a}^{ij}\text{,}\\ \max_{{\left(\phi_{i},\psi_{i}\right)}_{j=1}^{I}}\overset{I}{\underset{i=1}{\sum }}\overset{J}{\underset{j=1}{\sum }}\alpha_{1}L_{a}^{ij}+\alpha_{2}L_{r}^{i}+\alpha_{3}L_{d}^{ij}+\alpha_{4}L_{e}^{i}. \end{array}$$

In one training iteration, only one pair of generator-discriminators is updated rather than all generators and discriminators. In particular, a generator and a discriminator are randomly chosen and the loss is computed with a random batch of training data. Afterwards, for multiple generators and discriminators, the anomaly score of the sample **x**′ under test is

(11) $$\begin{array}{c} A\left(x^{\prime }\right)=\frac{1}{IJ}\overset{I}{\underset{i=1}{\sum }}\overset{J}{\underset{j=1}{\sum }}A\left(x_{i};\phi ,\psi ,\gamma \right)\text{.} \end{array}$$

The average of the outlier scores helps eliminate spurious scores if the model is not well trained on a particular test instance. The threshold *θ* is set to judge whether the test sample is abnormal as follows:

(12) $$\begin{array}{c} A\left(x^{\prime }\right)>\theta \text{.} \end{array}$$

## 3. Experiment

### 3.1. Experimental Data

In order to evaluate the qualitative and quantitative results of the proposed method and compare it with the state-of-the-art algorithms, this paper selects two public video anomaly detection datasets for experiments, namely, CUHK Avenue [14] and ShanghaiTech [15]. The CUHK Avenue dataset was filmed on the streets of the Chinese University of Hong Kong, which consists of 16 training and 21 testing videos collected from fixed scenes. The training normal data only include pedestrian walking, and there are 47 abnormal events including running and packet loss. Compared to the CUHK Avenue dataset, the ShanghaiTech dataset is very challenging and contains videos from 13 scenes with complex lighting conditions and camera angles. The total number of frames for training and testing reaches 274,000 and 42,000, respectively. The test set includes 130 abnormal events such as chases, quarrels, and sudden movements, which are scattered in 17,000 frames.

### 3.2. Evaluation Indicators

Based on previous work [14, 15], this paper adopts the area under the ROC curve (AUC) to evaluate the performance. The ROC curve is obtained by calculating the predicted anomaly score at each frame level by varying the threshold.

### 3.3. Experimental Setup

For both datasets, each frame of video is resized to 286 × 286, and video blocks of size 256 × 256 are randomly cropped during each iteration. The structure of the generator adopts C64 × (4 × 4)-C128 × (4 × 4)-C256 × (4 × 4)-C512 × (4 × 4)-C512 × (4 × 4)-DC256 × (4 × 4)-DC128 × (4 × 4)-DC64 × (4 × 4) structure. The first half is the encoder, and the second half is the decoder. The encoder first uses 64 convolutional layers with 4 × 4 convolution kernels and then uses 128 convolutional layers with 4 × 4 convolution kernels. The decoder and encoder structures are completely opposite and contain deconvolutional layers of the same size. The BatchNorm layer and the ReLU activation function are connected after each layer. The discriminator includes a total of 5 convolutional layers, and the size of the convolution kernel is also 4 × 4. The structure adopts C64 × (4 × 4)-Pooling-C128 × (4 × 4)-Pooling-C256 × (4 × 4)-Pooling-C512 × (4 × 4) and finally outputs one-dimensional data. This paper uses TensorFlow2.0 to implement the GAN ensemble method and uses the Adam optimizer (*ρ*_1_ = 0.9, *ρ*_2_ = 0.999) to optimize it. The initial learning rate is set to 1e−4 and decays by 0.8 after every 50 epochs, for a total of 300 epochs of training.

### 3.4. Experimental Results

In order to verify the advantages of the method proposed in this paper, it is compared with some existing methods, which are from different types. The first ones are based on density estimation and probability models including VEC [16] and Conv-VRNN [17]. The second ones are single-class classification-based methods including MNAD-P [18] and AMDN [19]. The third ones are reconstruction-based methods including Conv2D-AE [6] and StackRNN [20]. The comparative results are given in Table 1, and the results of other methods are obtained from related papers.

From Table 1, it can be observed that the GAN ensemble model proposed in this paper achieves better results than the state-of-the-art methods on both datasets, which proves the effectiveness of the proposed method. In particular, it achieves an AUC of 91.1% on the CUHK Avenue dataset. It is worth noting that the performance of these methods on CUHK Avenue dataset is better than that on ShanghaiTech dataset, which is due to the fact that ShanghaiTech is a newly proposed dataset with a large number of frames and a large variation in different sample resolutions. In spite of this, the method proposed in this paper achieves 75.1% frame-level AUC on the ShanghaiTech dataset, which also exceeds the best VEC [16] among other methods by 0.3%.

**Figure 2:**
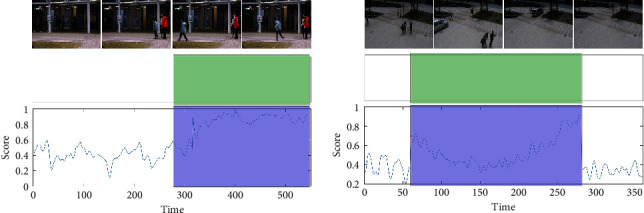
Two examples of anomaly detection comparison on CUHK Avenue dataset and ShanghaiTech dataset.

Figure 2 shows anomalous examples of the two test datasets for the proposed method. The anomaly curve shows the anomaly scores for all frames of the video in turn, through which the performance of the method can be observed more intuitively. The green area represents the anomalous part of the ground truth, and the blue area represents the abnormal area detected by the method. It can be seen that the blue area can correspond to the green area. In the normal frame part, the proposed GAN ensembles have low anomaly scores and are very stable. Also, when anomalies occur, such as bicycles and cars on the sidewalk, fights, and pushes, the anomaly score suddenly increases. The scores in the figure correspond exactly to the occurrence of these scenes. All the above results show that the proposed method can achieve superior results on video anomaly detection by comparison with some state-of-the-art methods.

## 4. Conclusion

This paper introduces ensemble learning into a GAN-based anomaly model for anomaly detection. The discriminator of GAN is very effective for anomaly detection, and ensemble learning can further improve the training of the discriminator. Therefore, the method proposed in this paper is not a simple combination of ensemble learning and GAN. The ensemble learning can effectively affect the prediction quality. Experiments on two datasets demonstrate that the proposed method outperforms some state-of-the-art methods for video anomaly detection. Extensive experiments show that the ensemble approach achieves superior results on both datasets compared to a single model [21, 22].

## Figures and Tables

**Table 1:** Comparison of frame-level anomaly detection performance with the state-of-the-art methods (AUC (%)).

Methods	CUHK Avenue	ShanghaiTech
VEC [16]	90.2	74.8
Conv-VRNN [17]	85.8	—
MNAD-P [18]	88.5	70.5
AMDN [19]	84.6	—
Conv2D-AE [6]	70.2	—
StackRNN [16]	80.9	68.0
Proposed	90.6	75.1

## Data Availability

The datasets used in this paper can be accessed upon request.
